# Update on known and emergent viruses affecting human male genital tract and fertility

**DOI:** 10.1186/s12610-024-00222-5

**Published:** 2024-03-14

**Authors:** Sara Dabizzi, Mario Maggi, Maria Gabriella Torcia

**Affiliations:** 1 Andrology, Women’s Endocrinology and Gender Incongruence Unit, Center for the Prevention, Diagnosis and Treatment of Infertility, Azienda Ospedaliera Universitaria Careggi Hospital, Florence, Italy; 2 Endocrinology Unit, Azienda Ospedaliera Universitaria Careggi Hospital, Florence, Italy; 3 Department of Experimental and Clinical Biomedical Sciences “Mario Serio”, University of Florence, Viale G. Pieraccini 6, Florence, Italy; 4 Department of Clinical and Experimental Medicine, University of Florence, Florence, Italy

**Keywords:** Male genital tract, Viruses, Male fertility, Sexual transmission, Semen parameters

## Abstract

Many viruses infect the male genital tract with harmful consequences at individual and population levels. In fact, viral infections may induce damage to different organs of the male genital tract (MGT), therefore compromising male fertility. The oxidative stress, induced during viral-mediated local and systemic inflammation, is responsible for testicular damage, compromising germinal and endocrine cell functions. A reduction in sperm count, motility, number of normal sperm and an increase in DNA fragmentation are all common findings in the course of viral infections that, however, generally regress after infection clearance. In some cases, however, viral shedding persists for a long time leading to unexpected sexual transmission, even after the disappearance of the viral load from the blood.

The recent outbreak of Zika and Ebola Virus evidenced how the MGT could represent a reservoir of dangerous emergent viruses and how new modalities of surveillance of survivors are strongly needed to limit viral transmission among the general population.

Here we reviewed the evidence concerning the presence of relevant viruses, including emergent and re-emergent, on the male genital tract, their route of entry, their adverse effects on male fertility and the pattern of viral shedding in the semen.

We also described laboratory strategies to reduce the risk of horizontal or vertical cross-infection in serodiscordant couples undergoing assisted reproductive technologies.

## Résumé

De nombreux virus infectent l’appareil génital masculin avec des conséquences néfastes au niveau individuel et de la population. En fait, les infections virales peuvent induire des dommages à différents organes de l’appareil génital masculin (AGM), compromettant ainsi la fertilité masculine. Le stress oxydatif, induit lors de l’inflammation locale et systémique à médiation virale, est responsable de lésions testiculaires, compromettant les fonctions des cellules germinales et endocrines. Une réduction de la concentration et de la motilité des spermatozoïdes, du nombre de spermatozoïdes normaux ainsi qu’une augmentation de la fragmentation de l’ADN, sont les résultats habituels retrouvés au cours des infections virales qui, cependant, régressent généralement après la clairance de l’infection. Dans certains cas, cependant, l’excrétion virale persiste pendant une longue période, ce qui entraîne une transmission sexuelle inattendue, même après la disparition de la charge virale sanguine.

La récente épidémie de Zika et d’Ebola a montré à quel point l’AGM pouvait représenter un réservoir de virus émergents dangereux, et à quel point de nouvelles modalités de surveillance des survivants sont fortement nécessaires pour limiter la transmission virale au sein de la population générale. Dans la présente revue, nous avons examiné les preuves concernant la présence de virus pertinents, y compris émergents et réémergents, dans l’appareil génital masculin, leur voie d’entrée, leurs effets néfastes sur la fertilité masculine et le modèle d’excrétion virale dans le sperme.

Nous avons également décrit des stratégies de laboratoire pour réduire le risque d’infection croisée horizontale ou verticale chez les couples sérodifférents qui ont recours à des techniques de procréation assistée.

**Mots-clés** Voies génitales masculines, Virus, Fertilité masculine, Transmission sexuelle, Paramètres du Sperme

## Introduction

### Effects of viral acute infection on male reproductive apparatus

To date, at least 30 viruses have been found in human reproductive apparatus and many of them can cause serious damage to testicular tissue finally impairing spermatogenesis [1]. Moreover, some viral infections raise concerns about the transmission to the sexual partners.

Some sexually transmitted viruses give rise to a localized infection of the male genital tract (MGT): these include Human Papilloma Virus (HPV) and Herpes Simplex Virus (HSV) that enter the MGT through the mucous membrane of the penis. Most viruses, however, including those that are sexually transmitted such as HIV, HBV, HTLV, disseminate into distinct part of the MGT through the systemic route.

The blood-testis barrier (BTB) isolates germ cells from potential deleterious effects of antibodies and immune reactions [2]. BTB plays an essential role in spermiogenesis, however it does not represent an efficient barrier against viruses; in fact, either germinal or accessory cells of the MGT can be infected. Testis inflammation (orchitis) is characterized by an inflammatory response with high concentration of proinflammatory mediators along with leukocyte infiltration into the seminiferous tubules [3]. The reactive oxygen species (ROS) usually represents the core mechanism of damage within the MGT [4]. ROS-driven oxidation is accelerated by polyunsaturated fatty acids of the plasma membranes of testicular and sperm cells, resulting in lipid peroxidation and loss of membrane integrity [5]. Thereafter, the nuclear chromatin is exposed to oxidative damage leading to DNA fragmentation and cell apoptosis of testicular and sperm cells [6]. This oxidative stress compromises every type of testicular cells, including Sertoli and Leydig cells, therefore affecting spermatogenesis, sperm maturation and hormonal production. All these factors could compromise male fertility [7, 8].

Viruses able to penetrate BTB could also pass the blood epidydimal barrier (BEB) and induce inflammation within the urethra, the prostate, and the epididymis itself. Hence, sperm cells are exposed to oxidative damage also during their transit throughout the epididymis, and this can further cause reduced sperm motility and reduced normal morphology.

A common consequence of acute testis inflammation with perturbation of BTB is the production of autoantibodies against germinal cells. The latter can further affect steroidogenesis, spermatogenesis and sperm motility as well as sperm-oocyte binding, finally leading to couple infertility [9–11]. This scenario can happen only when viruses affect males with a full sexual maturity [12].

Impaired fertility, which in many cases regresses after infection clearance, is not the only consequence of viral infections. MGT, and testis in particular, must be considered as an immune privileged site depending on i) the presence of BTB that segregate post meiotic and meiotic spermatocytes in the luminal compartment of the seminiferous tubules and ii) a tolerogenic environment necessary to avoid autoimmune reaction against germ cells. The presence of BEB helps to strengthen the immune privilege in the MGT. Unfortunately, the presence of both BTB and BEB also helps to protect viruses from the immune reaction and contributes to create a perfect site for viral persistence after the acute viremia phase. In the course of Zika Virus (ZIKV) and Human Immune Deficiency Virus (HIV) infection it has been observed that the amount of viral nucleic acids in the semen exceeds blood viremia, even in the acute phase of infection [13]. Two extremely dangerous viruses, causing hemorrhagic fever – i.e. the Ebola Virus (EBOV) and Marburg Virus (MARV) – can persist for a very long time in the MGT. In fact, proven sexual transmission of EBOV from long-time survivors after official recovery contributed to re-initiate a new transmission chain in 2014 in Guinea [14]. High concentration of viral particles in the semen are likely responsible for sexual transmission of hepatitis B (HBV) and retroviruses like HIV [3].

The viral persistence in MGT of dangerous, emergent, or re-emergent viruses (ZIKV, EBOV, West Nile Virus—WNV) represents a serious epidemiologic problem that needs to be deeply investigated. For this purpose, longitudinal studies with repeated sampling in infected individuals are necessary since viral semen shedding may be intermittent.

The persistence of dangerous viruses in MGT also may contribute to irreversible damage of infected tissues. In fact, viral persistence does not necessarily mean viral latency and even a slow viral replication may induce autophagy or apoptosis of germ and accessory cells [15].

However, viral infection is not the only threat to male fertility. Common antiviral drugs have also been shown to impair male fertility. Ribavirin inhibits the inosine monophosphate dehydrogenase, necessary for guanosine triphosphate synthesis and induces cellular apoptosis [16]. Other antiviral drugs (efavirenz, lamivudine, stavudine, nevirapine, and tenofovir) have been reported to possibly impair sperm function, by inducing atrophy of the seminiferous tubules [17].

The present review aims to provide a comprehensive understanding of the mechanisms by which viruses known to infect MGT may affect male reproduction.

Particular focus will be made on those viruses that can be sexually transmitted. We will also deal with viruses that, through sexual transmission, can infect the fetus. Finally, we will describe laboratory strategies aimed to reduce the risk of horizontal or vertical cross-infection in serodiscordant couples undergoing assisted reproduction technologies (ART). Table 1 summarize the main results for each virus.

**Table 1:** Viruses, taxonomy, effects on male reproductive tract, laboratory strategies of seminal fluid purification

*Virus*	*family*	*genome*	*Target organs in male apparatus*	*Affected sperm paramters*	*Effects on Pregnancy (natural or ART)/ newborn*	*Risk of transmission*	*Laboratory strategies*	*reference*
HPV	*Papillomaviridae*	DNA	epididymis, testicles, vas deferens, prostate and within the seminal fluid	sperm concentration, motility, morphology; increased sperm DNA fragmentation	reduced pregnancy rates, increased risk of miscarriage	To partner and collateral event on pregnancy	Sperm-washing/ swim-up technique with the addition of hyaluronidase	[18–26]
HSV	*Herpesviridae*	DNA	mucosal surface, nerve cells of peripheral ganglia, external genitalia MGT	low sperm count, increased viscosity, reduced sperm motility and apoptotic death	miscarriage	Vertical transmission	None: washing ineffective	[27–34]
HIV	*Retroviridae*	RNA	lymphocytes, macrophages and dendritic cells in prostate and seminal vesicles	volume, motility and morphology		To partner and collateral event on pregnancy	sequential density gradient, swim-up and washing followed by (RT)-PCR for viral genome	(https://www.who.int/health-topics/hiv-aids) [35–38]
HCMV	*Herpesviridae*	DNA		Sperm concentration and morphology	Severe neonatal disorders (hearing loss, severe neurological and sensorineural damage)	Vertical transmission	multiple washing or gradient separation methods	[30, 31, 39–47]
HBV	*HEPADNAviridae*	DNA	Testis cells (spermatogonia, spermatocytes, spermatids and sperms; Sertoli cells)	Motility and viability, mature sperms apoptosis, increased sperm DNA fragmentation		unlike	None	(https://www.who.int/health-topics/hepatitis) [48–53]
HCV	*Flaviviridae*	RNA		sperm count, motility and morphology	None (low fertilization rate in selected report)	To partner	sequential density gradient, swim-up and washing. Viral genome assessed by (RT)-PCR	[16, 53–62]
ZIKV	*Flaviviridae*	RNA	Testis (germ and Sertoli cells)	sperm concentration, motility, morphology	Congenital microcephaly, low birth weight, long-term effect on developing growth	Not reported	None: washing ineffective	[63–75]
EBOV	*Filoviridae*	RNA	testes, seminal vesicles, prostate, and bulbourethral glands	Not reported	Not reported	To partner	Not reported	[68, 76–79]
WNV	*Flaviviridae*	RNA		Not reported	Not reported	unlike	Not reported	[80–84]
MuV	*Paramyxoviridae*	RNA	Testis (Sertoli, Leydig and germinal cells)	sperm concentration, motility and morphology	Not reported	unlike	Not reported	[85–89]
SARS-CoV-2	*Coronaviridae*	RNA	Testis (germinal cells or in Sertoli and Leydig cells)	sperm concentration, progressive motility, morphology, DNA fragmentation	Not reported	Not reported	Not reported	[90–103]

### Survey methodology

First, the terms viruses and/or viral infections were searched in the PubMed database with different appropriate topics such as: semen, seminal fluid, male reproductive tract, fertilization. Abstracts were selected for relevance and the relative full-length publications were retrieved. Appropriate references of retrieved papers were studied and added when appropriated.

**Fig. 1:**
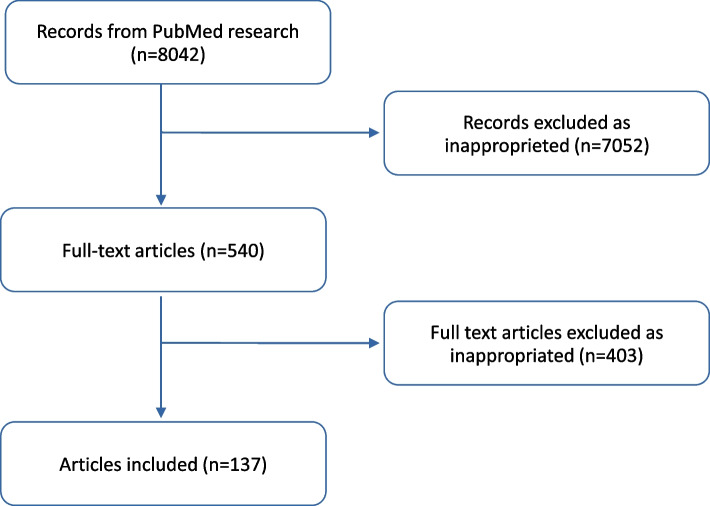
Flowchart for the systematic literature search

Second, the name of each single virus was searched in in the PubMed database with the topics mentioned above (semen, seminal fluid, male reproductive tract, Assisted Reproductive Technology – ART). Flow-chart of survey methodology is reported in Fig. 1.

### Viruses that induce localized infections in the MGT

#### Human Papilloma Virus (HPV)

##### General characteristic and target organs

Human Papilloma Viruses (HPVs) are DNA viruses belonging to the *Papillomaviridae* family that could infect the skin and mucosal surface of the anogenital area and of the upper respiratory tracts [104]. More than 200 HPV genotypes are known and were classified into high- and low-risk strains based on their oncogenic potential [105].

##### Epidemiology

HPV infection is often sexually transmitted, and the prevalence of male infection is similar to that of female, ranging from 3–45% [106].

##### Target organs in male

HPV infection in males is frequently associated with external genital warts, although asymptomatic infections are equally frequent. HPV was found at levels of epididymis, testicles, vas deferens, prostate and within the seminal fluid [18].

##### Impact on fertility

Numerous studies reported that high-risk HPV strains (HPV-16, HPV-18, HPV-31, HPV-33) may affect semen parameters, such as sperm concentration and motility, along with sperm morphology [19, 20]. The most common genotype in semen is HPV16, which belong to the group of high-risk HPV [21] and is associated to decrease of total sperm count.

Increased DNA fragmentation was also reported in the infected males [22] with possible, negative effects on reproductive capacity. Accordingly, a recent meta-analysis reported that the prevalence of HPV infection is significantly higher in infertile men compared to the general population (20.9% versus 8.2%) [23]. An increased risk of abortion and an overall reduced pregnancy rates were noted in HPV + patients undergoing ART treatments [24, 25], suggesting to include HPV testing in the European Tissue Cell Directive for couples attempting ART and to use sperm washing procedures and modified swim-up techniques in the attempt to avoid infected cells [24, 26].

##### Molecular mechanism

The molecular mechanisms involved in impairment of sperm parameters have not been elucidated yet. It is known that the virus binds to syndecan-I, a glycosaminoglycan located on the sperm head, through the L1 capsidic protein, and that this effect could reduce sperm motility [107]. Studies on infertile men demonstrated that patients with HPV infection had higher values of anti-spermatozoa antibodies (ASA) compared to a non-infected group [19, 107]. The role of ASA in reproduction is debated and different mechanisms have been proposed to explain reduced fertility, including sperm agglutination, impaired penetration of cervical mucus and complement activation throughout the female genital tract [19].

HPV infection is also associated to a lower capacity for oocytes fertilization and is closely related to miscarriages when using ART [25].

##### Screening and detection test

Nucleic Acid Tests (NAT) and PCR techniques on cytological sample (i.e. qPCR – broad detection range quantification and multiple detection—and ddPCR- simultaneous detection of different types and quantification of clinical load) allow to distinguish different HPV genotypes and to quantify of the viral load [108]. The most common genotype in semen is HPV16, which belong to the group of high-risk HPV [21].

Recently the presence of the virus in semen samples from men with high or low levels of risk was successfully detected by Nested PCR techniques that show high analytical sensitivity of lower viral load [109]. Nevertheless, molecular investigations via real time-PCR for detection of mRNA copies in seminal fluid attests the absence of potentially infected virions [109].

##### Risk of transmission

Recent reports documented sperm infection also in sexually active asymptomatic male [109]. An appropriate counseling on behavioral education of couples undergoing to natural or assisted reproduction may help to avoid the risk of transmission to partner and collateral event on pregnancy. In fact, in most cases, the presence of HPV in the semen is transient.

##### Risk in sperm banking

HPV testing is not currently mandatory for patients undergoing sperm banking and the risk of cross-contamination through liquid nitrogen is limited when high security straws are used. Nevertheless, the effect of the virus positivity in semen on fertility potential and the rapidly developing area confirm the importance of testing patients before banking [20].

##### Technical treatments

Sperm preparation techniques such as swim-up or sperm-wash can reduce the presence of virus on samples [24, 26], but cannot completely remove because HPV remains adherent to the sperm head.

An in vitro study shows that the most efficient technique to remove HPV from sperm is a modified swim-up technique with the addition of hyaluronidase to inhibit the HPV- sidecan-I interaction [18].

##### Vaccines

In HPV infected men, current HPV vaccines were reported to improve semen quality and to increase pregnancy rates in couples undergoing ART cycles [110].

#### Herpes simplex virus (HSV)

##### General characteristic and target organs

Herpes simplex virus (HSV) HSV-1 and HSV-2 are DNA viruses belonging to the *Herpesviridae* family that can be sexually transmitted. Genital HSV-1 and HSV-2 are largely diffused among the adult population. These viruses usually penetrate through genital ulcers, or because of penile epithelial trauma [27]. After replication in keratinocytes of the mucosal surface, the viruses reach nerve cells of peripheral ganglia through axonal transport and usually remain latent long-life.

Periodically, reactivation of virus induces the comparison of single or clustered vesicles on the surface of genital parts that usually undergo to ulceration before resolution. While primary infections may cause fever or localized adenopathy, the subsequent outbreaks are milder and resolve within a few days.

##### Epidemiology

Recent data from World Health Organization -WHO [111] report that globally 67% of people under age 50 has HSV-1 infection while 13% of people aged 15–49 worldwide has HSV-2 infection, the main cause of genital herpes. Both HSV-1 and HSV-2 have been detected in the semen of infected individuals with a viral shedding ranging between 0 and 100% in chronic infections [112]. HSV-2 infects all parts of the MGT [28] and strongly facilitates the HIV acquisition [29].

##### Impact on fertility

As reported above, the DNA of herpes viruses is frequently detected in the semen of asymptomatic fertile and infertile male patients. However, it is still unclear whether HSV1 and HSV2 play a role in male infertility. HSV infection has been associated with low sperm count, reduced sperm motility and apoptotic death [30, 31], as well as with abnormal viscosity, suggesting prostate inflammation [32].

Like many viruses, HSV might be transmitted vertically through the sperm cells and can increase the risk of miscarriage [33]. Molecular studies on identification of HSV in semen match the presence of HSV-1 in semen and lower mean sperm count, while the presence of HSV-2 is associated to lower seminal volume [31]. Instead, the correlation between the presence of HSV2 particles and sperm motility is weaker [31].

##### Infection pathways and technical treatments

HSV-2 particles were found both in seminal plasma and in spermatozoa, adhering to sperm membrane [31]. During ART procedures, even two-density-gradient washing method has been shown to be ineffective in reducing the viral load in semen [34], probably due to a strong binding of virus particles with sperm heads.

### Viruses that induce systemic infections

#### Human Immunodeficiency Virus (HIV)

##### General characteristic and target organs

HIV belongs to the family of *Retroviridae*. The virus infects CD4 + lymphocytes, macrophages and dendritic cells enriched in the submucosal compartment. Cell entry is mediated by the coordinate interaction of the viral envelope gp120 protein with the CD4 receptors and the co-receptor CCR5/CXCR4 which mediate viral entry. In the absence of anti-retroviral therapy, the infection leads to acquired immunodeficiency syndrome (AIDS).

##### Epidemiology

Thirty-nine million people live with HIV. In the last year 630 000 people died from HIV-related causes and 1.3 million people acquired HIV (https://www.who.int/health-topics/hiv-aids).

##### Infection pathways

Most HIV infections are sexually transmitted and semen represents the most important vector.

Semen contamination usually occurs during the stages of either acute or chronic infection and represents a crucial point for viral transmission and HIV epidemiology [35].

In the seminal fluid, the virus is present in at least three different forms: spermatozoa-associated virions, cell-free virions and infected leukocytes [36–38]. It is generally accepted that spermatozoa are not infected, although they behave as passive carriers of HIV, facilitating attachment to the cell surface. In this context, it has been reported that heparin sulphate proteoglycans facilitate virus attachment to the cell surface [54, 113]. Cell free virions or infected leukocytes play a more decisive role in sexual transmission [36].

##### Impact on fertility

Prostate and seminal vesicles are the main susceptible targets of HIV infection and represent the viral reservoirs that could release HIV into the seminal plasma [114].

Viral particles infecting seminal plasma, may induce infection of the testis. Evidence of orchitis is commonly present in male HIV infection and may result in hypergonadotropic hypogonadism with impaired steroidogenesis [115]. Reduction of ejaculated volume, sperm motility and morphology have also been reported in HIV + -patients [113, 116, 117]. The highly active antiretroviral therapy (HAART) does not solve these problems, since most of antiretroviral drugs do not cross the BTB [36] and the therapy *per se* may induce atrophy of seminiferous tubules and might impair spermatogenesis and steroidogenesis [118].

##### Technical procedures in ART

To avoid infection of the partner, serodiscordant couples should turn to in vitro procedures of ART. When male is infected, spermatozoa are isolated by sequential density gradient, swim-up techniques and washing to eliminate the fraction which contains infected leukocytes or cell-free virions. Then, to further reduce the risk of infecting the partner, the absence of viral genome is assessed by Real Time (RT)-PCR techniques [55]. This procedure is mandatory considering that, despite washing and gradient, evidence of minimal residual risk in the semen still exists [55].

##### Risk in sperm banking

HIV test is mandatory for sperm banking, by evaluation of HIV1/2 antibodies in blood. If result of test is positive, a separate handling and storage system must be set up [119].

#### Human cytomegalovirus (HCMV)

##### General characteristic

Human cytomegalovirus (HCMV) is a DNA virus belonging to *Herpesviridae* family that latently infects most of the general population. It causes a subclinical infection of the salivary glands in immunocompetent hosts that often persists throughout life. Reactivation of latent virus occurs in immunocompromised hosts resulting in significant morbidity and mortality. The virus has been detected in many biological fluids (blood, urine, feces, tears, saliva, breast milk, cervical mucus) including seminal fluid. Transmission of HCMV can occur in numerous ways: via blood transfusions, organ transplantation, breastfeeding, and sexual transmission.

Remarkably, HCMV infection can be vertically transferred during pregnancy [39]. In developing countries, congenital infection is present in 1–5% births [40]. It causes severe neonatal disorders, resulting in hearing loss and severe neurological and sensorineural damage [39]. Studies on the infection from HCMV in sub-fertile men report a wide variability in prevalence between 8 and 65% [33].

##### Epidemiology

Approximately 60% of the global population older than six years old has been exposed to HCMV. Seroprevalence in the population increases with age.

##### Impact on fertility

Numerous studies associate the presence of HCMV in the semen with alteration of seminal parameters, including concentration and morphology [41]. However, it is common opinion that such toxic effects are related to viral infiltration within the testis, rather than to the presence of virions in the semen. Indeed, a sensitive decrease of germinal cells with a gradual destruction of germinal epithelium was detected in histological testis explants of infected men [42].

HCMV infection can affect fertility through numerous pathways, mostly mediated by the host-inflammatory response. For example, inflammatory cytokines may affect the composition of the genital secretions with a consequent impact on the sperm-cervical mucus relationships. In addition, ASA can be produced as a result of virus-induced damage. Infection of Leydig cells can affect the endocrine function of the testicle [113, 115, 117]. Finally, HCMV infection, as other HSVs, may increase the risk of other sexually transmitted diseases, including HIV [43].

More controversial is the effect of HCMV on sperm quality parameters, including concentration, motility and morphology. Although some authors reported a correlation between the presence of HCMV in the semen and a decrease in sperm motility and concentration [30, 44], many others did not find any HCMV effect on sperm quality [45–47].

##### Risk of partner infection and technical treatments in ART

Mature sperms are potentially vectors of viral particles, either as products of infected germinal cells as well as for weak interaction reported between sperms and virions [112, 118]. Nonetheless, in animal models, no viral particles or HCMV-DNA has been reported in fertilized oocytes, blastocyst or fetal tissue [120]. Moreover, Lippold et al*.* demonstrated the presence in human seminal plasma of a heat-resistant factor that inhibits the attachment of the virus on cells surface in a dose-dependent manner and potentially can limit the viral transmission during sexual intercourse [121].

##### Technical treatments in ART

In ART procedures with a HCMV + male partner is highly recommended multiple washing or gradient separation methods to avoid the introduction of virus particles in ooplasm [31].

##### Risk in sperm banking

HCMV testing is not required both for partner and no partner sperm donor, but it is currently requested due to the high prevalence risk in population or if required by stricter national legislation. For test positive resulting, separate handling and banking procedures must be set up.

#### Hepatitis B Virus (HBV)

##### General characteristic and prevalence

Hepatitis B virus (HBV) is a DNA virus belonging to the *HEPADNA (hepatic DNA) viridae* family. HBV replicates into hepatic cells through an RNA intermediate and integrates into the host genome, a feature that confers the ability to persist in infected cells. The virus is most commonly transmitted from mother to child during birth and delivery, as well as through contact with blood or other body fluids (wounds with sharp instruments, unsafe injections).

Moreover, HBV is present as a free virus in the ejaculate [122]. The virus therefore is also sexually transmitted. Sexual transmission is an important route of dissemination in areas with a low or intermediate prevalence [48].

##### Epidemiology

The virus is largely diffused all over the world and is considered one of the most important viruses threatening global public health. Almost 296 million people were living with chronic hepatitis B infection in 2019 with 1.5 million new infections each year (https://www.who.int/health-topics/hepatitis).

HBV infection indeed persists in almost 5% of infected individuals and may evolve to hepatic cirrhosis and liver cell carcinoma [122]. The last report of WHO estimates that more than 800000 people died for HBV-related cirrhosis and hepatocellular carcinoma (primary liver cancer) in 2019.

##### Impact on fertility

HBV can cross the BTB and can infect and replicate into male germ cells following genome integration. Viral genome sequences were revealed by in situ hybridization techniques not only in germ cells—i.e. spermatogonia, spermatocytes, spermatids and sperms—but also in Sertoli cells [122]. The virus induces oxidative stress in infected germinal cells and ROS production, usually correlated to viral load, which is responsible for sperm apoptosis.

Accordingly, a larger number of apoptotic sperm cells was found in patients with chronic HBV infection compared with uninfected individuals. In vitro and in vivo studies have shown that, in mature sperms, HBV exposure induces early events of apoptosis, such as Ca2 + intracellular dysregulation, due to loss of mitochondrial membrane potential, caspase activation and increase in DNA fragmentation [49].

Other studies reported that the HBV capsidic-S protein induces a loss of sperm membrane integrity in a dose-dependent manner [49–51].

##### Fertility outcome in medical assisted reproduction

Despite significant differences in sperm motility and sperm viability, no negative effects on outcome of ART, as IVF (In Vitro Fertilization)/ICSI (Intra Cytoplasmic Sperm Injection) or IUI (Intra Uterine Insemination), was reported [52]. Moreover, the occurrence of vertical paternal transmission is considered unlikely, mostly in case of vaccinated female partner [53].

##### Risk in sperm banking

HBV test is mandatory for sperm banking, by evaluation of HBs-Ag and anti-HBc in blood. Testing for anti-HBs can be useful for determining antibody protection from vaccination [119].

#### Hepatitis C Virus (HCV)

##### General characteristic

Hepatitis C Virus (HCV) is an RNA-virus belonging to the *Flaviviridae* family that primarily affects the liver, inducing acute hepatitis.

Hepatitis C is mainly transmitted through contact with infected blood. Used needles or syringes, unsafe medical procedures and blood transfusions with unscreened blood products are responsible for most infections. Sexual transmission among heterosexual couples is very low [123]. In contrast, the virus however is easily transmitted during homosexual intercourse among male HIV-infected partners [124, 125].

Most HCV infections are usually asymptomatic and do not lead to a life-threatening disease. Symptoms of acute hepatitis are fever, fatigue, loss of appetite, nausea, vomiting, abdominal pain, dark urine and yellowing of the skin or eyes (jaundice).

The virus is spontaneously cleared in 30% of infected people within 6 months. The remaining 70% (55–85%) of persons will develop chronic HCV infection leading to serious liver disease, cirrhosis and cancer [53]. There is no vaccine for hepatitis C, but it can be treated with antiviral medications. Early detection and treatment can prevent serious liver damage and improve long-term health.

##### Epidemiology

HCV infection has world-wide diffusion and is often related to HIV infection.

The last report from WHO estimates that globally, 58 million people have chronic hepatitis C virus infection, with an incidence of 1.5 million new infections per year. Approximately 290 000 people died from hepatitis C in 2019, mostly from cirrhosis and hepatocellular carcinoma (https://www.who.int/health-topics/hepatitis).

##### Impact on fertility

HCV was found in the seminal plasma. The virus however is not able to integrate its genome or to replicate in the infected sperm [122, 126]. In addition, it was never found inside the spermatozoa or attached to their surface [33]. In infected patients, many studies reported altered sperm count, motility and morphology as well as reduction of fertility potential [16, 54, 56, 57]. High rate of oxidative stress could affect the permeability of mitochondrial membranes and could induce sperm chromatin condensation and apoptotic death of sperm cells [9].

Moreover, HCV may induce the development of an autoimmunity response, revealed by an ASA activation response [127].

Interestingly, even the treatment with appropriate therapies, such as ribavirin and interferon (IFN), might be responsible for the worsening of semen parameters [16, 57, 58, 128].

##### Screening and detection test

As above reported, HCV is found in the seminal plasma. Viral load in the seminal plasma can be measured by RT-PCR and, except for high viremia load [129], there is a poor correlation between blood and semen HCV load [125, 130].

##### Technical procedures in ART treatments

Contradictory results on the treatment outcome of male-affected couples in ART are reported in scientific literature. A lower fertilization rate is found in only a few cases [59, 60]. However, no significant difference in the final cycles outcome were evidenced [60, 61].

To reduce the risk of HCV transmission in ART, the guidelines recommend treating sperm from HCV + male with sequential washing, density gradient centrifugation and swim up, followed by RT-PCR to assess absence of viral genome [53, 55, 58, 62].

##### Risk in sperm banking

HCV test is mandatory for sperm banking, by evaluation of antibodies in blood. If the result of the test is positive, a separate handling and storage system must be set up [119].

#### Zika virus (ZIKV)

##### General characteristic

Zika virus (ZIKV) is an RNA virus belonging to the *Flaviviridae* family. ZIKV is mainly transmitted by Aedes mosquitos. The virus can be transmitted from mother to fetus during pregnancy and can induce fetal malformations in up 10% of cases with congenital microcephaly [11], low birth weight [63] as well as long-term effects on infant and developing growth [64]. ZIKV is also transmitted through transfusion of blood and blood products, through organ transplantation and by sexual transmission. WHO report indicates ZIKV as the first arbovirus associated with sexual partner transmission [65]. Viral RNA was found in seminal plasma either as free virions or as sperm-associated virus [66, 67], the presence revealed by reverse transcription polymerase chain reaction [68]. Several studies report that ZIKV is detected in the semen until the 188th day following infection [66, 69, 70]. High viral load and long-term ZIKV shedding in semen is not correlated to blood viral load [71], suggesting that the MGT is a possible reservoir of virus [72, 73], in which it can persist even without replicating [74] due to the restricted immunological response.

##### Risk of partner infection and technical procedures in ART treatments

Although there is no evidence of an offspring transmission by male paternal infection, couples are discouraged from planning pregnancy for almost three months after infection [55]. ART is also strongly discouraged since ZIKV was found in the semen even after sequential gradient centrifugation and selection by swim-up [75, 131]. Therefore, no additional treatments of semen sample are safe.

##### Target organs and impact on fertilizing potential

Testes appear to be the organ of choice for ZIKV replication: viral antigens were revealed in germ [66] and Sertoli cells [132]. Sertoli cells appear to be more susceptible to the infection than other testicular cells, due to the presence on their surface of the AXL receptor used by ZIKV to entry into the cells. ZIKV also induces an inflammatory response within the testis that potentially compromises spermatogenesis [73, 132, 133].

Accordingly, upon infection, many studies reported a temporary impairment of spermatogenesis with a relevant reduction of sperm concentration and motility [72, 74, 75] and with anomaly in head sperm morphology [75].

##### Risk in sperm banking

Screening for ZIKV is recommended depending on the donor’s history of travel or exposure. If it is not possible to postpone the procedure, separate storage and handling of infected samples are necessary [119].

#### Ebola virus (EBOV)

##### General characteristic

Ebola virus (EBOV) is single-stranded RNA virus belonging to the family of *Filoviridae*, which can lead to severe hemorrhagic fever in humans and other primates. Fruit bats of the *Pteropodidae* family represent the natural reservoir but the virus is present in numerous animals including primates. The virus is transmitted through direct contact with all body fluids. It is also present in semen in the acute phase of the disease and persist in MGT up to three months after the disease onset [76].

##### Epidemiology

Ebola virus infection was a sporadic, localized disease in West Africa. After The 2014 outbreak in West Africa, the Ebola virus disease is perceived as a widespread threat to public health in heavily populated regions [77]. The EBOV persistence in the semen of survivors is a frequent phenomenon and has contributed a lot to modify the epidemiology of the disease: exposure to seminal EBOV from male survivors is considered as possible cause of the resurgence of Ebola virus in 2021 in Guinea, seven years after the end of 2014 outbreak [78].

##### Impact on fertility

A positive relation between the age of infected man and the length of time in which virus RNA is revealed in the semen, is reported [68]. In experimental models, using non-human primates, the virus has been detected within the testes, seminal vesicles, prostate, and bulbourethral glands [79]. EBOV persists in Sertoli cells and induces the breakdown of the BTB with negative consequences on spermatogenesis as well as on the endocrine activity of the testis. EBOV induces a high production of pro-inflammatory cytokines and the relative oxidative stress associated to the inflammation is the main cause of cell and organ damage. Until now, the high mortality of the EBOV disease has been a limitation to the collection of data on the effects of EBOV on the genital apparatus and, in particular, on seminal fluid.

### Viruses that affect MGT but not relevant for sexual transmission

#### WEST NILE virus (WNV)

##### General characteristic

West Nile virus (WNV) is an enveloped single strand RNA virus belonging to the Flaviviridae family [80]. The virus is transmitted by mosquitos (culex genus) and can infect many birds’ species that represent the viral reservoir. The virus also infects mammals, including humans giving in a small percentage of cases (20%) fever, headache, vomiting, cutaneous rash. In less than 1% of infected people, an encephalitis can occur [81]. WNV indeed could invade the Central Nervous System (CNS) and other tissues, causing a strong pro-inflammatory cytokines response which reduces the functionality of Blood Brain Barrier (BBB) and allows the virus to enter the brain inducing encephalitis [82]. In patients with a neuroinvasive disease the mortality reaches 9%. Moreover, almost 50% of infected patients reported long-term neurological consequences after recovery and viral RNA was found in the brain or urine many months or years after from the acute illness [83].

WNV outbreak is seasonal in temperate zones with peaks from July to October in United States and Europe. Since the first recognition in 1999, the virus has spread in Africa, Middle East and Europe [68] following bird migration routes and being linked to temperate areas with higher temperature and precipitation in summer, excluding just Antarctic areas [81].

##### Impact on fertility and risk of partner transmission

The presence of WNV in the semen of infected individuals was rarely reported. Armah et al. reported the presence of WNV RNA in samples of testes and prostate of one out of four individuals deceased for neuroinvasive illness [84]. Another study [83] found WNV RNA in only one sample of semen collected after the onset of symptomatology. Nonetheless, although controversial, the possibility of sexual transmission was documented [68].

Actually, no updates regarding the impairment of male fertility were reported.

##### Technical procedures in ART treatments

Unfortunately, tests for viral detection on semen or plasma are not available yet and consequently neither efficient sperm washing procedures are reported. Nonetheless, international guidelines recommend screening of patients undergoing ART in the seasonal period between June and October, when the virus has maximum diffusion, by nucleic acid test (NAT) in plasma [68]. Whenever possible a delay of the assisted procedure is recommended.

##### Vaccines

There are currently no vaccines available and the only preventive measures concern the control of the vector mosquito [81].

#### Mumps Virus (MUV)

##### General characteristic

Mumps Virus (MuV) is an enveloped RNA virus belonging to the *Paramyxoviridae* family. It causes mild respiratory diseases in approximately 30% of infected people. The most salient feature of the infection is represented by parotid gland swelling. MuV infection can lead to inflammation of reproductive apparatus with orchitis and epididymites in almost 40% of males infected which occur about a week after the parotitis onset [12].

##### Vaccines

The introduction of the mumps virus (MuV) vaccine has greatly decreased the incidence of MuV infections.

Nonetheless, from 1999–2019, on average, about 500,000 mumps cases were reported to the World Health Organization annually. In 2021 ECDC reported 1567 cases of mumps (0.4 cases /100000 population) with a median age of 13 years.

##### Impact on fertility

MuV can infect Sertoli, Leydig and germinal cells. Sialic acid favors MuV entry into Sertoli and Leydig cells acting as a co-receptor. AXL and MER receptors have been suggested as further receptors or co-receptors for MuV [85]. The mechanisms underlying MuV-induced orchitis likely reside in a strong inflammatory response through Toll Like Receptor2 (TLR2) activation. IFN-type I production is induced by the cytosolic RIG-1 pathway and mediates innate response against MuV in Sertoli and Leydig cells. Infected germ cells, on the other hand, undergo autophagy [86].

In the acute phase of infection, the endocrine function of testis is compromised. Acute symptoms last only a couple of weeks but testicular atrophy can occur in almost 50% of infected patients and results in seminal abnormalities in sperm concentration, motility and morphology that could persist for years after recovery [87].

The presence of MuV in the semen has been reported until 14 days after infection [88]. Sexual transmission, however, is not relevant for the overall viral transmissibility.

#### Severe Acute Respiratory Syndrome Corona Virus 2 (SARS-CoV-2)

##### General characteristic

Sars-CoV-2 is a single-strand RNA virus belonging to *Coronaviridae* family that was responsible for the recent pandemic COVID 19 disease causing worldwide almost 800 million cases with almost 7 million deaths [89]. First variants spread all over the world caused mild to moderate respiratory illness although in some cases patients were seriously ill, requiring medical attention. Serious illness mostly occurred in older people or in people with severe chronic cardiovascular, chronic respiratory or endocrinological diseases (diabetes, obesity).

The virus potentially infects any cell expressing the Angiotensin Converting Enzyme 2 receptor (ACE2R) which interacts with the spike protein exposed on the viral envelope. Following ACE2R binding to spike protein, cellular proteolytic enzymes as furin and Trans Membrane Protease Serin 2 (TMPRSS2) allow the exposure of fusion fragment of spike protein and the consequent entry of virus into the cells [4, 90].

##### Impact on fertilizing potential

Many studies reported a significantly impaired sperm quality in COVID-19 infected patients, resulting in lower sperm concentration, reduced progressive motility, and increased morphological defects along with DNA fragmentation, as compared to pre-infectious parameters [91]. However, whether these effects are mediated directly by viral infection of the MGT is still controversial. ACE2 receptor is widely expressed in several testis districts, either in germinal cells or in Sertoli and Leydig cells [92]. ACE2R is expressed in epididymis epithelium and in some districts of the seminal vesicles [93]. TMPRSS2 is highly expressed in germinal cells as well as in prostatic tissue. Numerous studies evidenced the detrimental effects of SARS-CoV 2 infection on testicular functions. However, with only few exceptions [93, 94] infective virions or viral nucleic acids were neither found in the semen of infected men [90, 95–98], nor from asymptomatic individuals infected with SARS-CoV-2 [99].

It is current opinion that, in the course of systemic inflammation, the impairment of the BTB may allow the virus access to germinal compartment.

Accordingly, it was observed that SARS-COV-2, similarly to other RNA viruses, promotes an oxydo-inflammatory response with atrophy of the seminiferous tubules [4]. High fever and cytokine-induced inflammation with leukocyte infiltration highly contribute to induce the death of germ cells, to impair spermatogenesis, and, overall, to worse all seminal parameters [100].

Negative effects on seminal parameters of SARS-CoV2 infections were observed also in patients with mild COVID 19 diseases, up to four months after infections [91]. A recent study on patients with idiopathic infertility reported significant decrease in sperm concentration with high rates of oligospermia and asthenospermia up to six months after infection [101].

##### Risk of partner infection and fertility outcome

According to the absence of infective virions within the semen, sexual transmission and/or vertical transmission [102] were not reported.

### Conclusion and perspective

Today it is known that almost 30 viruses are released in the semen. These viruses include both viruses giving genital localized infection (HPV, HSV) and systemic viruses that can infect all organs of the MGT.

Male reproductive functions can be affected by damage of testis, epididymis and accessory glands induced by viral replication, local inflammation with oxidative stress. High fever and systemic inflammation are however also involved in MGT alterations [103].

Evidence is accumulating that some part of the MGT represent a possible reservoir of known and emerging viruses, including arthropod-borne and life-threating viruses (ZIKV, EBOV). These viruses in fact persist in the semen even after the systemic clearance and lead to sexual transmission for long time.

Screening for viruses in the seminal fluids is crucial to avoid transmission to the partner, when asymptomatic infection, potentially compromising pregnancy, is suspected. For this purpose, the improving and diffusion of simple diagnostic technologies is strongly recommended.

Semen washing and ART are extremely helpful to prevent vertical and horizontal virus transmission, particularly in chronic/persistent infection. The improvement of techniques leading to minimize the cross-contamination risk is fundamental.

## Data Availability

All data generated or analyzed during this study are included in this published article and its supplementary information files.

## Abbreviations

ART: Assisted reproduction technologies
BBB: Blood brain barrier
BEB: Blood epidydimal barrier
BTB: Blood-testis barrier
EBOV: Ebola Virus
HAART: Highly active antiretroviral therapy
HBV: Hepatitis B virus
HCMV: Human Cytomegalovirus
HCV: Hepatitis C Virus
HEPADNA: Hepatic DNA viridae family
HIV: Human Immunodeficiency virus
HPV: Human Papilloma Virus
HSV: Herpes Simplex Virus
HTLV: Human T cell leukemia virus
ICSI: Intra Cytoplasmic Sperm Injection
IUI: Intra Uterine Insemination
IVF: In Vitro Fertilization
MGT: Male genital tract
MuV: Mumps Virus
NAT: Nucleic Acid Test
ROS: Reactive oxygen species
Sars-Cov2: Severe Acute Respiratory Syndrome Corona Virus 2
WHO: World Health Organization
WNV: West Nile Virus
ZIKV: Zika Virus
